# Market concentration in the ACA individual marketplaces

**DOI:** 10.1093/haschl/qxaf199

**Published:** 2025-10-21

**Authors:** David M Anderson, Daniel Ludwinski, Sayeh Nikpay, Ezra Golberstein

**Affiliations:** Department of Health Services, Policy and Management, University of South Carolina, Columbia SC 29208, United States; Department of Economics, Oxford College of Emory University, Emory, GA, United States; Department of Health Policy and Management, University of Minnesota, Minneapolis, MN, United States; Department of Health Policy and Management, University of Minnesota, Minneapolis, MN, United States

**Keywords:** Affordable Care Act, health insurance, competition, market concentration, premiums

## Introduction

The Affordable Care Act (ACA) individual health insurance market rely on managed competition among private insurers to deliver health insurance to over 24 million Americans in 2025.^1^ Changes in competition between insurers are associated with changes in premium levels and affordability for subsidized and non-subsidized enrollees.^2^ More insurers in a given county may be associated with lower gross premiums and federal premium tax credit costs while also improving the possibility of enrollees matching to preferred products.^3^

The competitiveness of ACA markets has fluctuated as changes in expected profitability and regulatory predictability have prompted insurers to enter or exit.^4,5^ Prior research has focused on the number of insurers as the measure of competitiveness with evidence suggesting markets with three or more insurers were competitive leading to lower consumer costs through price competition.^6-8^ However, there is likely heterogeneity in the degree of market concentration of insurers as measured by Herfindahl-Hirschman Index (HHI), even among markets with the same number of insurers. Characterizing both the degree of competition and market concentration of insurers may provide future insight into premium levels and affordability in the ACA marketplaces.

This study describes the substantial variation in health insurer market power and concentration within the ACA individual markets between 2014 and 2023.

## Data and methods

Our primary enrollment data is from the 2014-2023 Issuer-Level Enrollment Data public use files published by the Centers for Medicare and Medicaid Services.^9^ We used the 2014-2023 Qualified Health Plan Landscape public use files to count on-exchange county-year plan participation for states that used Healthcare.gov.

We identified insurers using the five-digit HIOS ID. We calculate county-year HHI by first computing each insurer's market share, summing the square of those shares and multiplying by 10 000. For insurers whose county-year enrollment data was suppressed for privacy reasons, we acted as if the enrollment for these insurers was zero. Markets with an HHI of 1000-1800 are moderately concentrated; above 1800 is highly concentrated, while a market with a single insurer monopoly has an HHI of 10 000.^10^

We acknowledge several limitations. First, we only observe on-Exchange enrollment. Second, our data are limited to state-years on Healthcare.gov and excludes states using state-based marketplaces. Third, ACA health insurance products are differentiated on premiums, networks, and cost-sharing which may make interpreting the HHI less straightforward. Fourth, we measure HHI at county even as some insurers may offer partial county products. Finally, we likely underestimate HHI as we measure HHI by the number of unique 5 digit HIOS IDs instead of corporate parent which may control one or more unique HIOS IDs in a given county-year.

## Results

**Figure 1.**
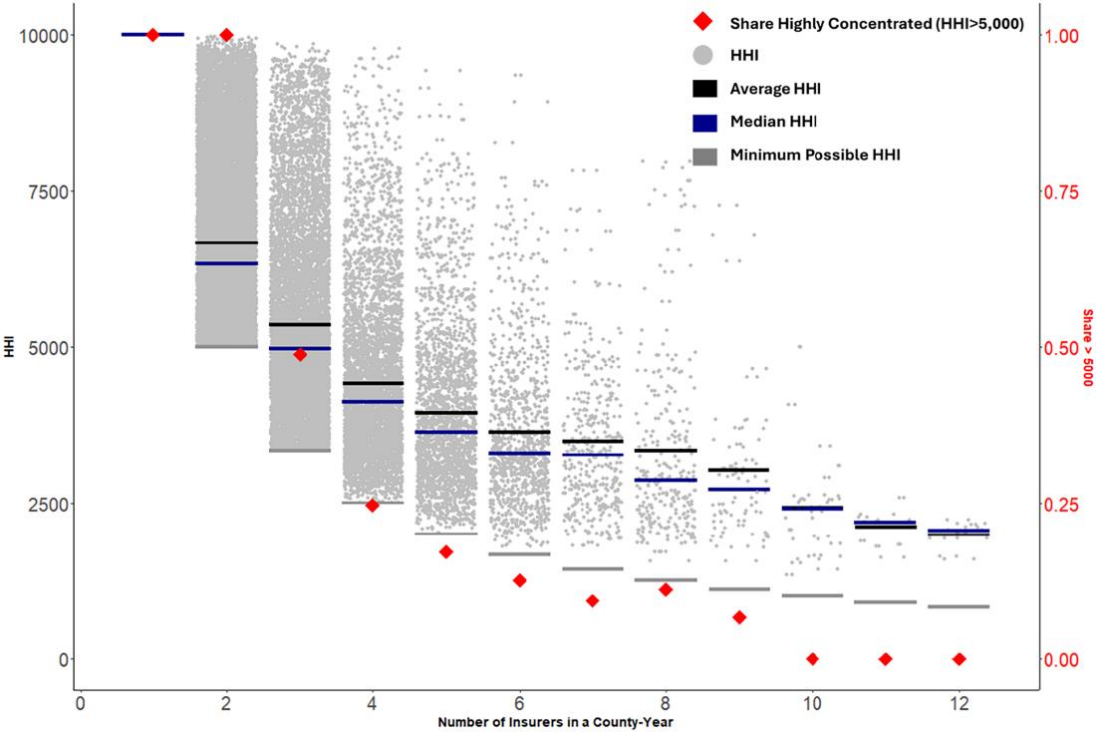
Variation in HHI in the ACA individual health insurance marketplaces 2014-2023 by the number of insurers. Source: Author's calculations from the 2014-2023 Issuer Level Enrollment Data and 2014-2023 QHP Landscape Public Use Files. The dark gray line is the mean HHI for the number of insurers in a county. The blue line is the median HHI by county-year-number of insurers. The light gray line is the minimum possible HHI for a given number of insurers in a county where each insurer had an equal market share.

On average, HHI is lower when there are more insurers in a county (Figure 1). However, within each category of insurer number-county-year, HHI varies substantially except for single-insurer counties where HHI is mechanically 10 000 (Figure 1). For example, in county-years with two insurers the interquartile range of HHI was 5354-7812. In county-years with four insurers the interquartile range of HHI was 3470-4978. And for county-years with six insurers the interquartile range was 2771-4175. We also find that a substantial share (76.2% total counties and 44.2% of population weighted county-years) of county-years have very-highly concentrated insurance markets (HHI > 5000). 19.9% county-years have at least four insurers and an HHI above 5000.

## Discussion

This study demonstrates wide variation in enrollment concentration in ACA insurance markets that have the same number of insurers. This study builds on earlier research which has either used insurer HHI or variations in insurer numbers by examining the degree of alignment between these measures, thereby contributing to a more comprehensive understanding of market power and competition within insurance markets. Future work examining the impact of multi-line of business market concentration and spillover effects of concentration using these data and other data sources should be pursued.

The ACA markets are likely to shrink in terms of enrollment and insurer participation in 2026 and beyond due to recent regulatory and legislative actions. The impacts of these market exits will likely vary by the size and market power of insurers. The ACA individual health insurance markets are a critical component of the American health insurance coverage landscape. Meaningful competition may lead to lower premiums, which results in lower federal subsidy costs per enrollee and improved affordability for non-subsidized enrollees. Policymakers, should pay differential attention to markets that are losing insurers, with more attention to markets that are already highly concentrated and likely to become even more highly concentrated. Markets should be evaluated for competitiveness both by the number of insurers and insurer enrollment concentration.

## Supplementary Material

qxaf199_Supplementary_Data
